# Effect of femtosecond laser-assisted cataract surgery for cataracts after pars plana vitrectomy: a prospective randomized controlled study

**DOI:** 10.1186/s12886-025-03871-w

**Published:** 2025-02-17

**Authors:** Lin Wen, Hao Lian, Yi Liu, Bin Wei, Yan Deng, Jianqi Hu, Ying Wu, Min Zhang, Yaoqin Fan, Li Xu

**Affiliations:** 1 Chengdu Aidi Eye Hospital, No. 45, West Section 2, Ring 2 Road, Qingyang District, Chengdu, Sichuan China; 2 Ophthalmology department, West China Hospital, Sichuan University, Chengdu, Sichuan China

**Keywords:** Pars plana vitrectomy, Cataract, Femtosecond laser, Phacoemulsification, Average phacoemulsification energy, Effective phacoemulsification time, Corneal endothelial cell density, Complication

## Abstract

**Objective:**

To compare the efficacy of femtosecond laser-assisted cataract surgery (FLACS) and conventional phacoemulsification surgery (CPS) in treating postvitrectomy cataracts.

**Methods:**

Prospective randomized controlled study. Patients who underwent cataract surgery after pars plana vitrectomy (PPV) were randomly divided into the FLACS group and the CPS group. Preoperative data of all patients were collected to compare intraoperative complications, average phacoemulsification energy (AVE), effective phacoemulsification time (EPT), postoperative intraocular pressure, corneal endothelial cell density (ECD), and best corrected visual acuity (BCVA).

**Results:**

A total of 92 eyes were included in the analysis, with 47 eyes in the FLACS group and 45 eyes in the CPS group. The intraoperative AVEs and EPTs in the FLACS group were both lower than those in the CPS group (*P* < 0.05). In the FLACS group, incomplete prechopping and incomplete capsulorhexis occurred in 3 eyes (3/47, 6.38%), and incomplete lens dislocation occurred in 1 eye (1/47, 2.13%). In the CPS group, incomplete lens dislocation occurred in 2 eyes (2/45, 4.44%), and anterior capsule tears occurred in 1 eye (1/45, 2.22%). There was no statistically significant difference in intraoperative complications between the two groups (*P* > 0.05). Postoperatively, intraocular pressure (IOP) was lower in both groups than preoperatively, and there was no statistically significant difference in intraocular pressure between the two groups at three months postsurgery (*P* > 0.05). Three months postoperatively, the ECD in the FLACS group was greater than that in the CPS group, with less average endothelial cell loss (ECL) than that in the CPS group (*P* < 0.01). The BCVA in both groups improved to varying degrees compared with the preoperative values, with the FLACS group performing better than the CPS group on the first postoperative day (*P* < 0.05). There was no statistically significant difference between the two groups at one week, one month, or three months postoperatively (all *P* > 0.05).

**Conclusion:**

FLACS is safe and effective for treating post-PPV cataracts and, compared with CPS, facilitates early postoperative recovery with no difference in final visual acuity. Residual silicone oil in the anterior chamber post-PPV may lead to certain specific outcomes for FLACS. Although it may not affect surgical results, it is still noteworthy.

## Introduction

Since pars plana vitrectomy (PPV) was introduced into clinical practice in the 1970s, continuous improvements and refinements in surgical equipment and techniques have made it a common surgical method for treating vitreoretinal diseases [1]. The widespread application of PPV, along with the use of silicone oil or gas tamponade, has made cataracts the most common complication after PPV [2]. Cataract surgery post-PPV is more challenging and more prone to complications. The incision used in conventional phacoemulsification surgery (CPS) is small and well sealed, effectively reducing the risk of low IOP. CPS combined with intraocular lens (IOL) implantation is the preferred surgical method for treating post-PPV cataracts. Since Nagy et al. [3] first applied femtosecond lasers to cataract surgery in 2009, femtosecond laser-assisted cataract surgery (FLACS) has been widely recognized for its precision, safety, and efficiency, making FLACS one of the preferred choices for cataract treatment today [4]. Currently, there are few reports on the use of FLACS for treating post-PPV cataracts in China. This study employs a prospective randomized controlled trial to analyze the outcomes of FLACS and CPS combined with IOL implantation in the treatment of post-PPV cataracts, assessing the effectiveness of FLACS in treating post-PPV cataracts.

## Materials and methods

### Study design

From January 2023 to June 2024, cataract patients who had undergone post-PPV and met the inclusion criteria of this study at Chengdu Aidi Eye Hospital were selected as the study subjects. All patients who underwent cataract surgery were fully informed about the two surgical methods, FLACS and CPS, including their costs, advantages, and disadvantages. After receiving a complete explanation of the study, they signed informed consent for surgery. The patients were randomly divided into two groups: the FLACS group, in which FLACS combined with IOL implantation was used; and the CPS group, in which the CPS combined with IOL implantation was used. The cost difference due to the surgical method is covered by research funding. This clinical study was approved by the Ethics Committee of Chengdu Aidi Eye Hospital and adheres to the Declaration of Helsinki. All patients provided informed consent and signed the informed consent form.

*The inclusion criteria* were as follows: age ≥ 18 years; previous history of PPV surgery, more than 6 months postoperative; presence of significant lens opacity, best corrected visual acuity (BCVA) below 0.4 logMAR; anterior segment condition meeting the surgical requirements of FLACS; preoperative examination showing no definite macular edema (DME) or retinal redetachment; and ability to actively cooperate to complete femtosecond laser operation.

*The exclusion criteria* for patients were as follows: PPV surgery due to ocular trauma or recurrent retinal detachment; a history of corneal refractive surgery; silicone oil eye post-PPV; presence of significant silicone oil in the anterior chamber; obvious lens ectopia; intraocular pressure (IOP) > 21 mmHg not controlled; and cataract postoperative follow-up shorter than three months.

### Preoperative examination and preparation

The baseline characteristics and demographic details were recorded. All patients underwent routine preoperative examinations, including uncorrected distance visual acuity (UCVA) and BCVA, IOP (Topcon CT-80 A, Japan), slit-lamp and fundus examinations, anterior segment photography, corneal topography, corneal endothelial cell count (Sowin SW-7000, China), A/B ultrasound (Sowin SW-2100, China), IOLMaster 700 (Zeiss, Germany), macular OCT (Spectralis HRA + OCT, Germany), and wide-angle fundus photography (Daytona (P200T), UK). The axial length (AL) and IOL diopter were measured via the IOLMaster 700. If measurement was not possible, A/B ultrasound measurement was used. A foldable intraocular lens was selected, and the target diopter was set between − 0.30 D and − 3.00 D on the basis of the patient’s needs and AL.

### Surgical methods and medication

All surgeries were performed by the same experienced cataract surgeon via the same phacoemulsification machine (Bausch & Lomb BL11110, USA).

The control group was subjected to the CPS method. After pupil dilation, patients were placed in a supine position. Proparacaine hydrochloride eye drops (S.A. ALCON-COUVREURN. V, Belgium) were used for topical anesthesia. After the eyelid speculum was applied, the conjunctival sac was disinfected with 0.5% povidone-iodine. A side incision and a 2.2 mm main incision were routinely made. Medical sodium hyaluronate gel (Bloomage Bio, China) was injected into the anterior chamber. If necessary, indocyanine green injection (Dandong Medical Innovation, China) was used for anterior capsule staining. CCC capsulorhexis was performed, with a diameter of approximately 5.2 ∼ 5.5 mm. Thorough hydrodissection and hydrodelineation were conducted. The phacoemulsification machine parameters were personalized on the basis of the axial length and anterior chamber stability. The lens nucleus was emulsified and aspirated, and the lens cortex was irrigated and aspirated. The posterior capsule was polished, and a foldable intraocular lens was implanted into the capsular bag. The medical sodium hyaluronate gel in the anterior chamber and capsular bag was aspirated, and the incisions were watertight. Tobramycin and dexamethasone ophthalmic ointment (ALCON CUSI s.a., Spain) was applied to the conjunctival sac, and the operated eye was bandaged.

The FLACS method was used for the observation group. After pupil dilation, the eye was routinely disinfected, and the Catalys ophthalmic femtosecond laser system (Johnson & Johnson Vision, USA) was used to perform capsulorhexis, prechopping, and incision creation. The parameters were set to a capsulorhexis diameter of 5.2 ∼ 5.5 mm, centered relative to the capsule bag. An appropriate prechopping mode was selected on the basis of the hardness of the lens nucleus. After the operation, the eye was reinfected under a surgical microscope. After the eyelid was opened with a speculum, the conjunctival sac was disinfected with 0.5% povidone-iodine. The side and main incisions were separated, and medical sodium hyaluronate gel was injected into the anterior chamber. The cut anterior capsule membrane was removed via a manual capsulorhexis technique. For incomplete capsulorhexis, a secondary capsulorhexis was performed. The remaining steps were the same as those for the control group.

#### Perioperative medication

Preoperative levofloxacin eye drops (Santen-China, China) and pranoprofen eye drops (Senju Pharmaceutical Co., Ltd., Japan) were used. Postoperatively, tobramycin and dexamethasone eye drops were tapered weekly and combined with pranoprofen eye drops for 1 month. If the postoperative IOP was ≥ 25 mmHg, fluid release from an auxiliary incision or combined with intraocular pressure-lowering drugs was used to control IOP. The patients were subsequently followed up on postoperative days 1, 1 week, 1 month, and 3 months.

### Observation indicators

All preoperative and postoperative examinations were performed by the same personnel using the same equipment. The following preoperative data were collected from all patients: age, sex, lens nucleus hardness (using the Emery-Little classification), BCVA, IOP, corneal endothelial cell density (ECD), AL, intraoperative complications, average phacoemulsification energy (AVE), effective phacoemulsification time (EPT), BCVA at 1 day, 1 week, 1 month, and 3 months postoperatively, and IOP and ECD 3 months postoperatively. BCVA after Nd: YAG laser (LPULSA SYL-9000, Taiwan, China) treatment was included in the statistics for those with posterior capsule plaque or posterior capsular opacification (PCO) at least 1 month postoperatively.

### Statistical methods

Statistical analysis was carried out via the Statistical Package for Social Sciences (SPSS 19.0 version). Cross-tabulation chi-square tests were used for sex, lens nucleus hardness grading, primary disease conditions, and intraoperative and postoperative complications. Measurement data that met the homogeneity of variance criteria are expressed as the means ± standard deviations (means ± SDs). Independent samples t-tests were used for age, ECD, IOP, AL, AVE, and EPT. The preoperative and postoperative BCVAs of the two groups were compared via the Wilcoxon rank-sum test. Statistical significance was set at *P* < 0.05 for all tests.

## Results

### Basic information

There were 115 patients (115 eyes) who underwent cataract surgery after PPV, with 23 eyes excluded from the study. Among them, 8 eyes had significant silicone oil residue in the anterior chamber, 6 eyes were silicone oil-filled, 5 eyes had posterior synechia or pupil dilation < 6 mm, 3 eyes were lost to follow-up postoperatively, and 1 eye underwent ECCE surgery due to abnormal zonules during the operation. Finally, 92 patients (92 eyes) were included in the analysis, with 47 eyes in the FLACS group and 45 eyes in the CPS group. The basic information of the two groups is shown in Table 1. There were no statistically significant differences between the FLACS group and the CPS group in terms of age, sex, lens nucleus hardness grade, preoperative BCVA, ECD, AL, or IOP (*P* > 0.05). Among the primary diseases leading to PPV surgery in the two patient groups, the FLACS group had 36 eyes with rhegmatogenous retinal detachment (RRD), 7 eyes with epiretinal membrane, 3 eyes with diabetic retinopathy, and 1 eye with macular hole. In the CPS group, the primary diseases included 33 eyes with RRD, 7 eyes with diabetic retinopathy, 4 eyes with macular hole, and 1 eye with epiretinal membrane. There was a statistically significant difference in the primary diseases between the two groups (*P* < 0.05).

**Table 1:** Basic preoperative data of the FLACS and CPS groups

	FLACS group (*n* = 47)	CPS group (*n* = 45)	*P* value
Age (year)	53.34 ± 8.44	52.80 ± 13.84	0.823
Sex			0.848
Male	21	21	
Female	26	24	
Emery-Little Nuclear cataract grade			0.981
Grade I	0	0	
Grade II	8	9	
Grade III	19	18	
Grade IV	16	14	
Grade V	4	4	
BCVA(logMAR)			0.428
< 1	37	34	
< 0.5	10	9	
< 0.3	0	2	
The mean ECD (cells/mm^2^)	2561.87 ± 397.85	2658.31 ± 311.89	0.200
AL(mm)	26.49 ± 2.61	27.28 ± 3.39	0.212
IOP(mmHg)	15.54 ± 3.43	15.68 ± 3.34	0.841
Protopathy			0.046
RRD	36	33	
Diabetic retinopathy	3	7	
Macula hole	1	4	
Epiretinal membrane	7	1	

FLACS: femtosecond laser-assisted cataract surgery; CPS: conventional phacoemulsification; BCVA: best corrected visual acuity; logMAR: log of minimum angle of resolution; ECD: endothelial cell density; AL: axial length; IOP: intraocular pressure; RRD: rhegmatogenous retinal detachment

### Surgical outcomes

For those with ALs ≥ 26 mm who met the criteria, capsular tension rings (CTRs) were routinely implanted, and IOLs were implanted within the capsular bag in all eyes. In the FLACS group, incomplete prechopping and capsulorhexis occurred in 3 eyes (3/47, 6.38%), and incomplete lens dislocation occurred in 1 eye (1/47, 2.13%). In the CPS group, incomplete lens dislocation occurred in 2 eyes (2/45, 4.44%), and anterior capsule tears occurred in 1 eye (1/45, 2.22%). There was no significant difference in the incidence of intraoperative complications between the two groups (*P* > 0.05) (Table 2). Neither group experienced severe intraoperative complications, such as nucleus drop, expulsive choroidal hemorrhage, or retinal detachment.

**Table 2:** Intraoperative and postoperative complications (eyes) in the FLACS and CPS groups

	FLACS group (*n* = 47)	CPS group (*n* = 45)	*P* value
**Intraoperative complications**			0.093
Presplit nuclei are incomplete	3	/	
Not completely torn	3	/	
Incomplete dislocation	1	2	
Radial tear of the anterior capsule	0	1	
**Corneal edema**	11	20	0.033
**Posterior capsule plaques or PCO**	16	15	0.943

FLACS: femtosecond laser-assisted cataract surgery; CPS: conventional phacoemulsification; PCO: posterior capsular opacification

### Postoperative conditions and management

On the first day after surgery, corneal edema occurred in 11 eyes (11/47, 23.04%) in the FLACS group and 20 eyes (20/45, 44.44%) in the CPS group, with a statistically significant difference (*P* < 0.05). The frequency of tobramycin and dexamethasone eye drops was increased, and for eyes with intraocular pressure ≥ 25 mmHg, fluid release from an auxiliary incision or combined treatment with intraocular pressure-lowering drugs was administered. The corneal edema subsided within 1 week postoperatively. Intraoperative findings of posterior capsule plaques or posterior capsule opacification during follow-up were observed in 16 eyes (16/47, 34.04%) in the FLACS group and 15 eyes (15/45, 33.33%) in the CPS group, with no statistically significant difference (*P* > 0.05) (Table 2). For posterior capsule plaques or PCOs affecting vision, Nd: YAG laser posterior capsulotomy was performed 1 month postoperatively.

### AVE and EPT

The AVEs and EPTs were recorded at the end of each surgery. Compared with that in the CPS group, the AVE in the FLACS group was 20.64% lower, and the EPT was 35.91% lower; these differences were statistically significant (*P* < 0.05) (Table 3).

**Table 3:** Intraoperative AVEs and EPTs in the FLACS and CPS groups

	AVE(%)	EPT(s)
FLACS group (*n* = 47)	12.15 ± 6.16	5.96 ± 4.82
CPS group (*n* = 45)	15.31 ± 7.28	9.30 ± 7.33
*t* value	-2.254	-2.578
*P* value	0.027	0.012

FLACS: femtosecond laser-assisted cataract surgery; CPS: conventional phacoemulsification; AVE: average phacoemulsification energy; EPT: effective phacoemulsification time

### Postoperative IOP and ECD

In the early postoperative period, some patients experienced corneal edema and an uneven distribution of corneal endothelial cells, leading to inaccurate IOP and mean ECD results. Therefore, the results of IOP and the mean ECD 3 months postoperatively were compared. Compared with the preoperative values, both the FLACS and CPS groups presented varying degrees of IOP reduction, with no statistically significant difference in IOP between the two groups (*P* > 0.05). The mean ECD of the FLACS group 3 months postoperatively was greater than that of the CPS group, with the mean endothelial cell loss (ECL) being significantly lower than that of the CPS group (*P* < 0.01) (Table 4).

**Table 4:** IOP and ECD three months after surgery in the FLACS and CPS groups

	IOP (mmHg)	ECD (cells/mm^2^)	ECL (cells/mm^2^)
FLACS group (*n* = 47)	13.53 ± 2.64	2185.57 ± 422.11	376.30 ± 275.55
CPS group (*n* = 45)	14.04 ± 2.65	1959.80 ± 348.00	698.51 ± 326.51
*t* value	-0.929	2.804	-5.123
*P* value	0.355	0.006	0.000

FLACS: femtosecond laser-assisted cataract surgery; CPS: conventional phacoemulsification; IOP: intraocular pressure; ECD: endothelial cell density; ECL: endothelial cell loss

### BCVA

The distributions of BCVA during the follow-up period for the FLACS group and the CPS group are shown in Table 5. Both groups showed varying degrees of improvement compared with before surgery. On the first day after surgery, the FLACS group was significantly superior to the CPS group (*P* < 0.05), whereas at 1 week, 1 month, and 3 months after surgery, there was no statistically significant difference between the two groups (all *P* > 0.05).

**Table 5:** Postoperative BCVA(logMAR) in the FLACS and CPS groups(eyes)

group	1 d				1 w				1 mo				3 mo			
	< 1	< 0.5	< 0.3	≥ 0.3	< 1	< 0.5	< 0.3	≥ 0.3	< 1	< 0.5	< 0.3	≥ 0.3	< 1	< 0.5	< 0.3	≥ 0.3
FLACS group (*n* = 47)	14	15	10	8	8	15	10	14	6	13	9	19	5	14	7	21
CPS group (*n* = 45)	16	17	11	1	11	16	9	9	7	15	11	12	6	15	9	15
*Z* value	-1.983				-1.192				-1.122				-0.978			
*P* value	0.047				0.233				0.262				0.328			

FLACS: femtosecond laser-assisted cataract surgery; CPS: conventional phacoemulsification; BCVA: best corrected visual acuity; logMAR: log of minimum angle of resolution

## Discussion

The pathogenesis of cataract formation after PPV surgery is still unclear and may be related to multiple inducing factors [5–8]. The most common type of cataract after PPV surgery is nuclear sclerosis, with an incidence rate of 81% within 6 months and as high as 100% within 2 years [5–6, 8]. In this study, all lenses in the eyes showed varying degrees of nuclear sclerosis, with 75 eyes (75/92, 81.52%) having a lens nucleus graded as Emery-Little grade III or above and 38 eyes having hard nuclear cataracts at Grade IV or above, accounting for as many as 41.30% (38/92). In this study, the proportion of post-PPV hard nuclear cataracts was relatively high, possibly because (1) post-PPV visual function decreased to varying degrees, causing patients to focus more on retinal recovery and neglecting the impact of lens opacification; (2) poor compliance in some patients led to a prolonged interval before silicone oil removal, and prolonged silicone oil tamponade accelerated lens nucleus hardening; and (3) patients with insufficient postoperative follow-up time failed to detect lens clouding at an early stage, and by the time of long-term consultation, lens nucleus hardening was already very severe.

A newly discovered special suspensory ligament inserts from the posterior insertion area of the vitreous zonule through the ciliary body pars plana and connects to the equatorial posterior capsule of the lens [9]. Together with the vitreous zonule and the traditional lens suspensory ligament, it forms the lens suspensory ligament system, which stabilizes and regulates lens movement. The surgical channel of the PPV passes through the ciliary body pars plana, and in the process of clearing the anterior vitreous, it inevitably leads to varying degrees of damage to the lens suspensory ligament system. The abnormal anatomical structure around the lens post-PPV, as well as lens nucleus hardening, increases the difficulty of cataract surgery. The anterior chamber deepens after post-PPV cataracts, and the poor fundus red reflex in hard nuclear cataracts increases the difficulty of capsulorhexis. Compared with manual capsulorhexis, FLACS yields better capsulorhexis results and better postoperative IOL centration [10]. The degree of lens nucleus sclerosis is related to the energy of phacoemulsification and the operation time, and FLACS, through prechopping, can effectively reduce the use of ultrasound energy. Abell RG et al. [11] reported that in routine cataract surgery, femtosecond laser prechopping treatment can significantly reduce EPT, potentially achieving 0 EPT. Asif MI et al. [12] analyzed 60 eyes with LOCS III nuclear grading ≤ NO4 cataracts, and the average CDE in the FLACS group was 48.8% lower than that in the CPS group (*P* = 0.012), with the average ECD being better than that in the CPS group (*P* = 0.001). Cai L et al. [13] analyzed 86 cataract eyes (45 in the FLACS group and 41 in the CPS group), including 43 with hard nuclei. The results revealed that FLACS patients with hard nuclei had a lower average CDE, shorter ultrasound time, lower average ECL, and faster recovery of central corneal thickness than CPS patients with hard nuclei. Notably, the proportion of silicone oil filling after PPV surgery is relatively high. Emulsified silicone oil can enter the anterior chamber through the zonular space. During manual capsulorhexis, viscoelastic agents can be used to push away the silicone oil in the anterior chamber, but the silicone oil in the anterior chamber affects the penetration of the femtosecond laser, reducing its effectiveness. This study rigorously excluded eyes with significant emulsified silicone oil residue in the anterior chamber. However, in the FLACS group, there were still three eyes with small amounts of emulsified silicone oil in the angle, partially covering the pupillary area during femtosecond laser treatment, resulting in incomplete prechopping and incomplete capsulorhexis. In the FLACS group, 3 eyes (3/47, 6.38%) had incomplete capsulorhexis, all of which were less than one clock hour in range. After manual capsulorhexis completion, no anterior capsule tears occurred. In the CPS group, 1 eye experienced anterior capsule tear (1/45, 2.22%). Using FLACS did not increase the risk of capsulorhexis complications. Incomplete capsulorhexis caused by silicone oil is often accompanied by incomplete prechopping. The 3 eyes with incomplete prechopping and capsulorhexis in the FLACS group were all the same eye, which is also a unique intraoperative complication of FLACS. Although there were 3 eyes with incomplete prechopping in the FLACS group, after prechopping, the AVE and EPT in the FLACS group were still significantly lower than those in the CPS group (*P* < 0.05). Compared with that in the CPS group, the AVE in the FLACS group was reduced by 20.64%, and the EPT was reduced by 35.91%, indicating that when post-PPV cataracts are treated with FLACS, even in the presence of incomplete prechopping, ultrasound time and ultrasound energy can still be significantly reduced. In the FLACS group, there was one case of incomplete lens dislocation, and in the CPS group, there were two cases. After implantation of the capsular tension ring, successful in-the-bag IOL implantation was achieved in all patients. There was no significant difference in intraoperative complications between the two groups (*P* > 0.05). No severe complications, such as expulsive choroidal hemorrhage or retinal detachment, occurred intraoperatively in either group.

Ultrasound time and energy are important factors affecting ECD. When FLACS reduces the ultrasound time and energy, it can effectively reduce the loss of corneal endothelial cells and alleviate corneal edema. Abell RG et al. [11] reported that pretreatment with a femtosecond laser can reduce the average ECL by 36.1%. Krarup T et al. [14] followed patients with one eye who underwent FLACS surgery and the other eye who underwent CPS surgery for 6 months. Compared with the CPS group, the FLACS group presented a 30% reduction in average ECL on day 40 and a 21% reduction on day 180. In this study, the proportion of hard nuclear cataracts with Emery grade IV and above was high (38/92, 41.30%), with prolonged ultrasound time and higher ultrasound energy, which is one of the reasons for the high proportion of postoperative corneal edema (31/92, 33.70%). On the first day after surgery, corneal edema occurred in 11 eyes (11/47, 23.40%) in the FLACS group and in 20 eyes (20/45, 44.44%) in the CPS group, which was a significant difference (*P* < 0.05). Three months postoperatively, the ECD in the FLACS group was greater than that in the CPS group, with an average ECL loss of less than that in the CPS group (*P* < 0.01), resulting in 46.13% less loss than that in the CPS group. During surgery, we combined the use of dispersive and cohesive viscoelastics, employing the soft shell technique to protect corneal endothelial cells, further reducing the trauma caused by ultrasound energy to corneal endothelial cells. Noor NA et al. [15] utilized the anterior capsule flap created in FLACS to provide additional protection for corneal endothelial cells, especially in eyes with a lower ECD, which is beneficial for reducing the average ECL. In clinical practice, various techniques can be combined to reduce the impact of ultrasound energy on corneal endothelial cells.

The good capsulorhexis results of FLACS can reduce IOL tilt and decentration, decrease aberrations, and improve postoperative visual acuity and quality [16]. Chee SP et al. [17] reported that the more precise size, roundness, and centration of capsulorhexis achieved with FLACS result in superior postoperative UAVA compared with CPS. Asif MI et al. [12] reported that in a study focusing on cataracts with LOCS III nuclear grading ≤ NO4, there was no difference in postoperative corrected visual acuity between the FLACS group and the CPS group. In a prospective study by Ewe SY et al. [18], patients with better economic means underwent FLACS surgery earlier, resulting in baseline BCVA for FLACS being superior to that of CPS, and BCVA at 6 months postoperative was also superior to that of CPS. Some studies have reported a greater chance of early posterior capsule opacification after FLACS [19]. We observed that in some patients, the lens posterior capsule had already developed plaque-like opacification post-PPV. These opacifications may not be completely removed during cataract surgery. Therefore, for posterior capsule plaques or opacifications affecting vision, we performed Nd: YAG laser capsulotomy at least one month after cataract surgery and used the posttreatment BCVA for statistical analysis. In this study, the postoperative BCVA of both groups improved to varying degrees compared with the preoperative BCVA. On the first day after surgery, the BCVA of the FLACS group was better than that of the CPS group (*P* < 0.05), which was related to the different degrees of corneal edema in the two groups of patients in the early postoperative period. As the degree of corneal edema subsided, the visual acuity of both groups gradually improved, and there was no significant difference in BCVA between the two groups at one week, one month, or three months postoperatively (*P* > 0.05), which is consistent with the results of early studies [12–13, 18].

FLACS reduces the difficulty of cataract surgery after PPV and minimizes the impact on corneal endothelial cells, benefiting both surgeons and patients. However, compared with CPS, FLACS is not cost-effective [20]. In our study, the cost of FLACS was almost twice that of CPS, and FLACS did not provide superior final visual acuity. Patients cannot directly perceive the advantages of FLACS, emphasizing the need for clinicians to select the appropriate surgical method on the basis of patients’ needs and practical situations. This study has several limitations, including a small sample size and relatively short follow-up duration, which precludes the evaluation of long-term outcomes and complications of FLACS for cataracts after PPV.

## Conclusion

In summary, compared with CPS, FLACS can effectively reduce ultrasound energy and time, providing patients with faster visual improvement and promoting early postoperative recovery. FLACS is safe and effective in treating post-PPV cataracts. Although there is no difference in final vision, it can be considered a new option for treating post-PPV cataracts. The presence of residual silicone oil in the anterior chamber after PPV surgery may lead to specific outcomes in FLACS. Although it may not affect the surgical results, it is still worth noting.

## Data Availability

The datasets used and/or analysed during the current study are available from the corresponding author on reasonable request.

## Abbreviations

FLACS: Femtosecond laser-assisted cataract surgery
CPS: Conventional phacoemulsification
PPV: Pars plana vitrectomy
AVE: Average phacoemulsification energy
EPT: Effective phacoemulsification time
ECD: Endothelial cell density
BCVA: Best corrected visual acuity
ECL: Endothelial cell loss
IOL: Intraocular lens
logMAR: Log of minimum angle of resolution
DME: Definite macular edema
IOP: Intraocular pressure
UCVA: Uncorrected distance visual acuity
AL: Axial length
RRD: Rhegmatogenous retinal detachment
PCO: Posterior capsular opacification
CTR: Capsular tension ring
